# The Pollution Status of Heavy Metals in the Surface Seawater and Sediments of the Tianjin Coastal Area, North China

**DOI:** 10.3390/ijerph182111243

**Published:** 2021-10-26

**Authors:** Xuemeng Han, Junqiang Wang, Wenqian Cai, Xiangqin Xu, Mingdong Sun

**Affiliations:** 1 College of Marine and Environmental Sciences, Tianjin University of Science & Technology, Tianjin 300457, China; hxm12135@163.com; 2 State Key Laboratory of Environmental Criteria and Risk Assessment, Chinese Research Academy of Environmental Sciences, Beijing 100012, China; wangjunqiang@tcare-mee.cn (J.W.); xiangxiang8696@126.com (X.X.); 3 Technical Centre for Soil, Agriculture and Rural Ecology and Environment, Ministry of Ecology and Environment, Beijing 100012, China

**Keywords:** heavy metals, pollution status, ecological risk level, Tianjin coastal area

## Abstract

Heavy metal pollution has become a great concern due to its adverse effects on the ecological system and human health. The present study investigated the concentrations of six common heavy metals (Cr, Cu, Zn, As, Cd, and Pb) in the Tianjin coastal area to understand their distribution, enrichment, sources, and potential ecological risk levels, focusing on the main contributors. The results showed that the concentration of Cu was high in the surface seawater (6.89 µg/L for the average), while Cd was the main contaminating metal in the sediments, with an average concentration of 0.77 mg/kg. The potential ecological risk index (*RI*) implied that the heavy metals in the sediments could cause considerable ecological risk, and Cd was the major contributor to ecological risk in this area. In particular, the field investigation showed that Cd contamination occurred as a result of anthropogenic activities, including port transportation, mariculture, and metal fabrication along the coastal area. Therefore, it is necessary to control Cd contamination in the future to improve the quality of the marine environment in Bohai Bay.

## 1. Introduction

In the aquatic ecosystem, heavy metal pollution has caused great concern across the world because of its high bioaccumulation, toxicity, and persistence [1,2,3,4,5]. It can exist for a long time in various environmental mediums [6,7] and, thus, can cause adverse effects on aquatic ecosystems and even threaten human life [8,9,10]. In general, anthropogenic activities contribute significantly to the concentration of heavy metals that are present in sediments above their natural background levels due to intensive human activities resulting from rapid social and economic development [5,11,12,13,14,15]. In the aquatic environment, as a sink for the majority of metal pollutants that are discharged into the sea, sediments have widely used as an environmental indicator to assess metal pollution status [16,17,18]. However, heavy metals are not permanently fixed in the sediment; disturbances caused by tidal action and the river flow can cause the secondary contamination of the overlying water and aquatic organisms in estuaries [12,19,20]. Hence, analyzing the heavy metals in surface water and sediments will help us to understand the variability of heavy metals in the marine ecosystem, which is helpful for the local governments to prevent and control the ecological risk caused by various heavy metals.

Due to their superior natural conditions, coastal areas tend to become centers of population and economic activities; therefore, they are also the main sink areas for pollutants, including heavy metals [19,20]. The Tianjin coastal area, which is located in Bohai Bay (Northern China), is a shallow basin with a very gentle slope, and the mean water depth is approximately 10 m [21]. Due to its hydrodynamic and oceanographic characteristics, the aquatic ecosystem in the Tianjin coastal area is more sensitive to human activities. Due to economic development in the Tianjin coastal area, pollutants have accumulated in this area for many years, resulting in a negative effect on the water quality [22,23,24]. However, previous studies have mainly focused on the drainage basin sediments of the Tianjin coastal area but seldom evaluated the concentration of heavy metals in the surface seawater and sediments of this area.

Therefore, this study aimed to (1) analyze the spatial temporal distribution of heavy metal (Cr, Cu, Zn, As, Cd, and Pb) concentrations in the surface seawater and sediments of the Tianjin coastal area in Bohai Bay; (2) assess the contamination and environmental risks of heavy metals in sediments using the *EF*, *I_geo_*, and *RI* indices; and (3) identify the potential sources of heavy metal contamination using multivariate analyses.

## 2. Materials and Methods

### 2.1. Sample Collection

**Figure 1:**
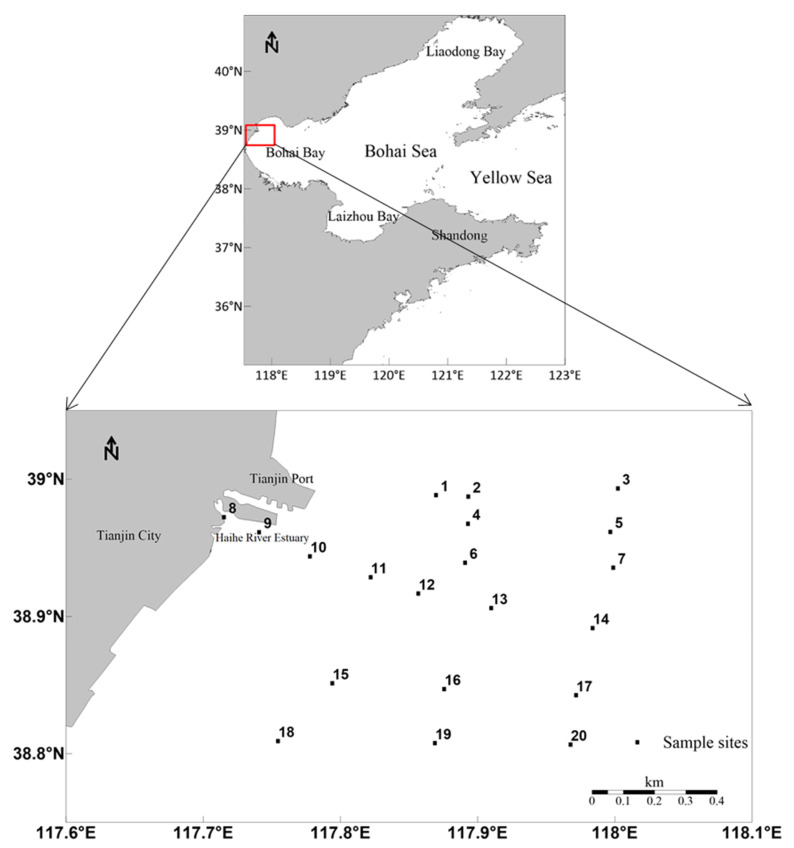
Sampling sites in the Tianjin coastal area, Bohai Bay, China.

The study area was the Tianjin coastal area, Bohai Bay, North China (Figure 1). A total of 60 surface seawater samples and 80 surface sediment samples were collected from 20 sites in May and September of 2011, September of 2012, and September of 2015 (Table S1). Surface seawater (at approximately 0.5 m depth) samples were collected using a Niskin water sampler. The water samples were filtered with a pre-cleaned cellulose membrane filter (0.45 μm pore size), adjusted to pH 2.0 using ultrapure nitric acid, and preserved at 4 °C until analysis. Sediment samples were collected with a grab sampler according to the ISO 5667–12 method [25]. Samples were transported to the laboratory at 4 °C in zippered plastic bags and tested as soon as possible.

### 2.2. Metal Analysis

For the water samples, the filtered water was analyzed directly. For the sediment samples, the sediments were freeze-dried, ground to pass through a 0.15 mm nylon mesh, and digested with a mixture of concentrated hydrochloric acid and nitric acid. The concentrations of seven metal elements (Cr, Cu, Zn, Pb, Cd, As, and Fe) in the aqua extracts and six metal elements (Cr, Cu, Zn, Pb, Cd, and As) in the water were determined using inductively coupled plasma mass spectrometry (ICP-MS, Agilent 7500 Series, Japan). Quality control measures were taken by carrying out reagent blank analyses in parallel with the samples. Samples were analyzed in triplicate, and the relative standard deviation (RSD) was in the range of 0.3–11%. The recovery rates of the seven metals in the spiked samples ranged from 80% to 95%. The method detection limits (MDLs) were 0.003, 0.01, 0.01, 0.003, 0.001, 0.05, and 0.03 µg/L for Cr, Cu, Zn, Pb, Cd, Fe, and As, respectively. In this study, Fe was used to calculating the EF value; the other six heavy metals were employed to analysis the pollution status.

### 2.3. Pollution Indices and Ecological Risk Assessment

Multiple approaches have been developed and widely used to assess the pollution status and ecological risk of metals in aquatic sediments [26,27,28]. The background concentrations of heavy metals in the sediments were 60, 26, 75, 15, 0.1, and 17 mg/kg for Cr, Cu, Zn, As, Cd, and Pb, respectively [29,30], and 2.92% for Fe [31]. These background concentrations were used for assessing the enrichment factor (*EF*), geo-accumulation index (*I_geo_*), and potential ecological risk factor (*RI*).

#### 2.3.1. Enrichment Factor

The enrichment factor (*EF*) was applied to assess the pollution degree of heavy metals in sediments that were impacted by anthropogenic activities following Equation (1) [32,33]. To minimize the variability of the sediment profile due to the influence of mineralogy and grain size, the metal concentrations of the sample were initially normalized by conservative elements, such as Fe, Co, As, and Sc. In this study, Fe was used as the normalization element.

(1) $$EF=\frac{C_{m}^{i}/Fe_{m}}{C_{bkgd}^{i}/Fe_{bkgd}}$$

where $C_{m}^{i}$ and $C_{bkgd}^{i}$ are the concentrations of each detected heavy metal element in the samples and their corresponding baseline value; $Fe_{m}$ and $Fe_{bkgd}$ are the concentrations of Fe in samples and its corresponding baseline value, respectively.

The pollution degree of heavy metals in sediments could be divided into six grades according to the *EF* value: no enrichment (<1.5), slight enrichment (1.5–2), moderate enrichment (2–5), severe enrichment (5–20), highly severe enrichment (20–40), and extremely severe enrichment (>40) [34].

#### 2.3.2. Geo-Accumulation Index (*I_geo_*)

The geo-accumulation index (*I_geo_*) was used to evaluate heavy metal pollution by eliminating the influence of geological factors [35], using Equation (2):

(2) $$I_{geo}=\log_{2}[\frac{C_{m}^{i}}{1.5*C_{bkgd}^{i}}]$$

where factor 1.5 refers to the background matrix correction value to weaken the lithogenic effect. Seven pollution level categories were devised based on the *I_geo_*: practically unpolluted (<0); unpolluted to moderately polluted (0–1); moderately polluted (1–2); moderately to strongly polluted (2–3); strongly polluted (3–4); strongly to extremely polluted (4–5); extremely polluted (>5) [36].

#### 2.3.3. Potential Ecological Risk Index

The potential ecological risk index (*RI*) proposed by Hakanson [28] could be calculated according to the following equations:

(3) $$E_{i}=T_{i}*\frac{C_{m}^{i}}{C_{bkgd}^{i}}$$

(4) $$RI=\sum_{i=1}^{n}E_{i}$$

where $C_{m}^{i}/C_{bkgd}^{i}$ is the contamination factor of a single heavy metal, and *E_i_* and *T_i_* are the potential ecological risk factor and the toxic response factor for a given metal, respectively. The toxicity response factors for Cr, Cu, Zn, As, Pb, and Cd were 2, 5, 1, 10, 5, and 30, respectively. The following terminologies are suggested for the *E_i_* and *RI* values: (1) *E_i_* < 40, low ecological risk; 40 < *E_i_* ≤ 80, moderate ecological risk; 80 < *E_i_* ≤ 160, high ecological risk; 160 < *E_i_* ≤ 320, very high ecological risk; *E_i_* > 320, serious ecological risk; (2) *RI* < 150, low ecological risk; 150 < *RI* < 300, moderate ecological risk; 300 < *RI* < 600, considerable ecological risk; *RI* > 600, significantly high ecological risk [37,38].

### 2.4. Statistical Analysis

The concentration of heavy metals in the surface seawater and sediment samples was statistically analyzed using SPSS 25.0 (International Business Machine, Beijing, China). Pearson correlation analysis and dual clustering analysis were performed to evaluate the statistical relationship, while the potential sources of heavy metal elements were determined using the R software (Version 3.6.1, freeware). The figures were drawn using the SURFER 15.0 (Golden Software, Golden, CO, USA). and R software (R Core Team, Vienna, Austria).

## 3. Results and Discussion

### 3.1. Distribution of Heavy Metal Concentrations in Surface Seawater and Sediments

#### 3.1.1. Seawater

The concentrations of heavy metals in the surface seawater of the Tianjin coastal area are presented in Table 1. The heavy metal concentrations during the study period were as follows: 0.09–0.67 µg/L for Cr, 1.25–17.08 µg/L for Cu, ND–69.57 µg/L for Zn, 0.81–12.53 µg/L for As, 0.07–0.43 µg/L for Cd, and 0.06–7.68 µg/L for Pb. The concentrations of Cr, As, and Cd were lower than the Grade I standard. Dissolved Cu, Zn, and Pb were the major pollutants in the surface seawater of the Tianjin coastal area, with the average concentrations being above the Grade I standard for the seawater quality standard in China [39]. The concentration values of Cu in 18 samples (30% of all samples) were between the limits of the Grade II and Grade III standards, which should be paid more attention, as this may potentially harm the environment. Additionally, this might pose a certain ecological risk for the living organisms in Bohai Bay, as the biological toxicity of Cu was reported to be high in the ocean [40].

Previous studies have shown that Cu tended to accumulate in the seawater, thus inducing higher concentrations of Cu in the surface seawater [41]. Moreover, in this study, the Cu concentration was generally higher than it is in other sea areas, with the exception of Jinzhou Bay (Table 2), which receives more heavy metal pollutants via the river input than Bohai Bay [42]. For example, 31 and 6 tons of heavy metal from the Daliaohe River and Shuangtaizi River, respectively, were discharged into Liaodong Bay in 2012, which is higher than the land-based pollutants from Tianjin city (19 tons) [43].

**Figure 2:**
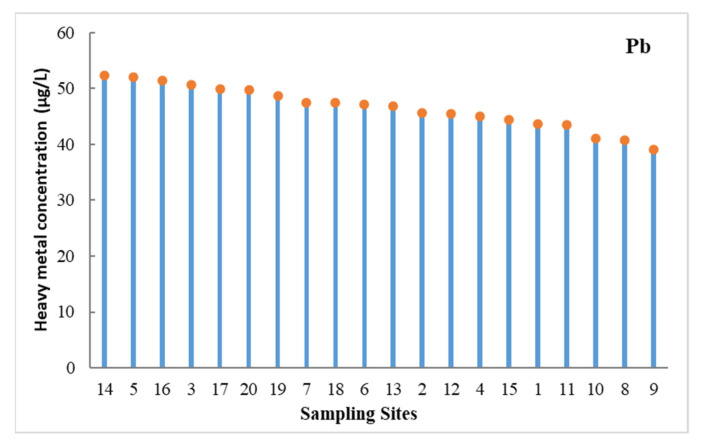
Concentrations of Pb in the surface seawater.

No obvious spatial distribution patterns were found in the dissolved Cr, Cd, Cu, Zn, or As concentrations in the surface seawater of the study area. The concentration of Pb increased in the open area compared to the coastal areas, with higher concentrations measured in the open area (Figure 2). It has previously been verified that atmospheric deposition is the main cause of the Pb contamination in Bohai Bay, inducing the higher Pb concentrations in the open area [22,48,49].

#### 3.1.2. Sediments

The heavy metal concentrations in the sediments ranged from 4.64 to 77.12 mg/kg for Cr, 1.70 to 75.94 mg/kg for Cu, 5.93 to 185.39 mg/kg for Zn, 0.87 to 53.49 mg/kg for As, 0.16 to 2.63 mg/kg for Cd, and 3.29 to 31.81 mg/kg for Pb (Table 3). Among all heavy metals, the average concentration of Cd was 0.77 mg/kg, which is higher than the Grade I marine sediment quality for China [50]. In particular, the Cd concentrations in 17 samples (21% of all samples) were between the values of the Grade II and Grade III standards. Moreover, the average concentration of Cd was higher than that in other sea areas (Table 4). This phenomenon could be related to the higher Cd background value in Bohai Sea [51]. Meanwhile, the rapid industrialization and urbanization in the Tianjin coastal area are an important source of heavy metal pollutants, including Cd [52].

**Figure 3:**
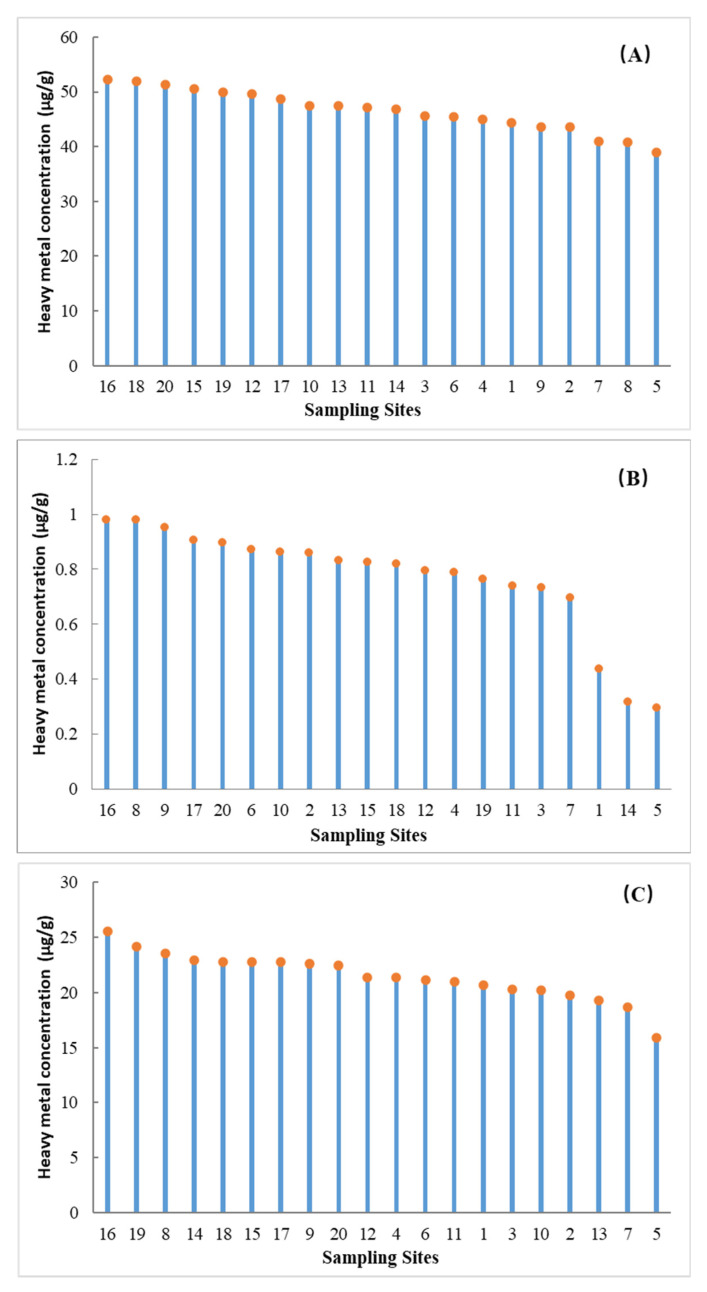
Concentrations of Cr (**A**), Cd (**B**), and Pb (**C**) in the sediments.

In general, the concentrations of Cr, Cu, Zn, Pb, and As in the sediments of the Tianjin coastal area were the highest in 2011 and the lowest in 2015. On the other hand, the opposite phenomenon was observed for Cd concentrations in the above three sampling years (Table 3), which showed a high agreement with the variation pattern of the land-based discharge (Table 5). Otherwise, the higher Cd and Pb concentrations were observed at the Haihe River estuary (Figure 3), indicating that they were mainly influenced by the river input pollutants from the neighboring metropolitan city [7,61]. Moreover, higher concentrations of Cd, Pb, and Cr were observed in the south of the study area (Figure 3), which could be a result of the higher sewage outfalls from the Ziyaxin River in the south area than from the other rivers in the area of this study, as indicated by previous studies [62].

### 3.2. Assessment of Heavy Metal Contamination

#### 3.2.1. Enrichment Status Assessment

**Figure 4:**
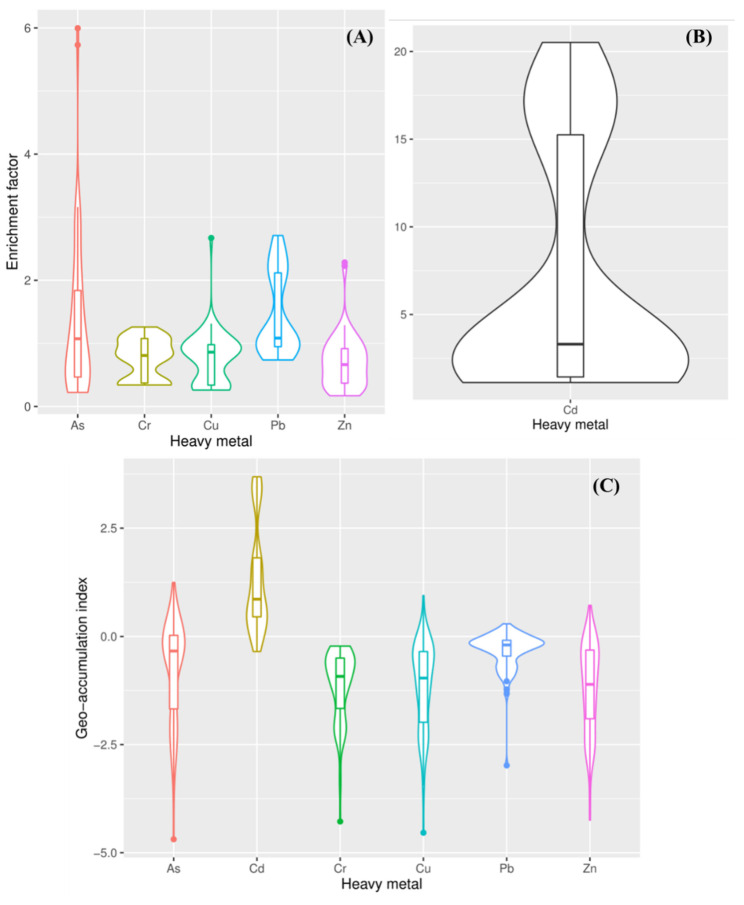
The *EF* (**A**,**B**) and *I_geo_* (**C**) values of heavy metals in sediments (the box represents the 25th to 75th percentiles; the solid line in the box represents the median value).

The mean *EF* values of heavy metals were ranked as follows: Cd (7.02) > Pb (1.46) > As (1.32) > Cu (0.78) > Cr (0.76) > Zn (0.71). The *EF* values were lower than 1.5 for Cu, Cr, and Zn in all samples, indicating no enrichment (Table S2). Over 50% of the *EF* values were lower than 1.5 for As and Pb (Figure 4), indicating no enrichment in the study area. Moreover, 38% of the samples were identified as being moderately enriched (2 < *EF* < 5) by Cd, and 28% of the samples were identified as being severely enriched (5 < *EF* < 20). The *EF* values of Cd showed an obvious decreasing gradient from the inshore area to the open sea area, with the highest values found at the Haihe River estuary (Figure S1). It shows that Cd in the sediments of the study area was affected by land-based discharges produced by anthropogenic activities.

#### 3.2.2. Assessment by Geo-Accumulation Index

The values of *I_geo_* ranged from −4.28 to −0.22 for Cr, −4.54 to 0.95 for Cu, −4.25 to 0.72 for Zn, −4.69 to 1.25 for As, −0.35 to 3.69 for Cd, and −2.98 to 0.29 for Pb (Table S3), with the rank of average values as follows: Cd (1.33) > Pb (−0.33) > As (−0.75) > Cr (−1.14) = Cu (−1.14) > Zn (−1.20). This is similar to the trend that was observed for the *EF* values. More than 90% of the samples for Cu, Zn, As, Cr, and Pb had *I_geo_* values that were lower than 0 (Figure 4), indicating the unpolluted status based on the contamination degree standard. In addition, 70% of the samples were identified as unpolluted to moderately polluted (0 < *I_geo_* < 1) and moderately polluted (1 < *I_geo_* < 2) by Cd, while 20% (distributed in the nearshore and estuary regions) were identified as strongly polluted (3 < *I_geo_* < 4) (Figure S1). In conclusion, the pollution status of Cd in the sediments was more serious than the other heavy metals in this study area, as was found to be the case in the coastal sediments of Bohai Sea [66].

#### 3.2.3. Risk Levels Assessment

The *E_i_* values for these heavy metals ranked in the following order: Cd (170.51) > As (11.49) > Pb (6.20) > Cu (4.18) > Cr (1.55) > Zn (0.81) (Table S3). The *E_i_* values of Cr, Cu, Zn, As, and Pb were lower than 40 in all of the samples, indicating the low potential ecological risk. However, the *E_i_* value of Cd was the highest among all six heavy metals in this study, with an average value of >160, indicating a much higher potential ecological risk. Moreover, approximately 15% of the samples had *RI* values ranging from 150 to 300, indicating a moderate ecological risk level. Additionally, approximately 17% of the samples ranged from 300 to 600, indicating a considerable ecological risk level (Table S3).

**Figure 5:**
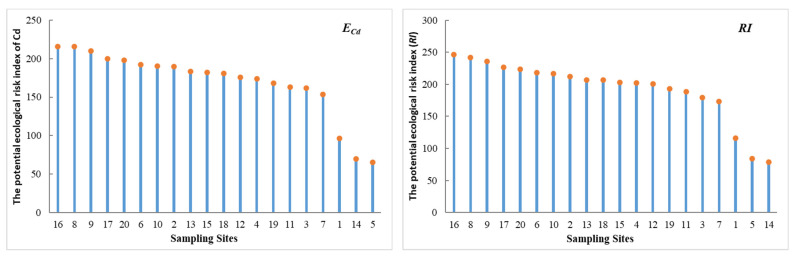
The values of *E_Cd_* and *RI* in the Tianjin coastal area.

A similar spatial distribution pattern was found between the potential ecological risk index of Cd and the *RI* values, both of which had higher values concentrated around the Haihe river estuary and the south part of the study area (Figure 5), reflecting the effects of industrial wastewater discharge into the sea [35]. At the same time, the *RI* values in the sediments of Tianjin coastal area were the highest in 2015 and the lowest in 2011 (Table S3), which was consistent with the Cd concentration trend in the sediments above (Table 4). As well, it was also consistent with the variation pattern of the land-based discharge (Table 5), indicating that it was affected by anthropogenic activities. As concluded by *RI*, *EF*, and *I_geo_* above, we can conclude that Cd made an important contribution to ecological risks in this study area. The high Cd concentration could be a serious threat to the aquatic ecosystem [67]. Furthermore, seafood species living in the aquatic ecosystem, such as scallops, could rapidly accumulate Cd from the surrounding environment, which could then threaten human health through the food chain [68]. Therefore, it is necessary to improve the monitoring of Cd concentrations in the sediments of Bohai Bay and strengthen the control of heavy metal emission amount to prevent an ecological crisis.

### 3.3. Source Apportionment of the Heavy Metals

**Figure 6:**
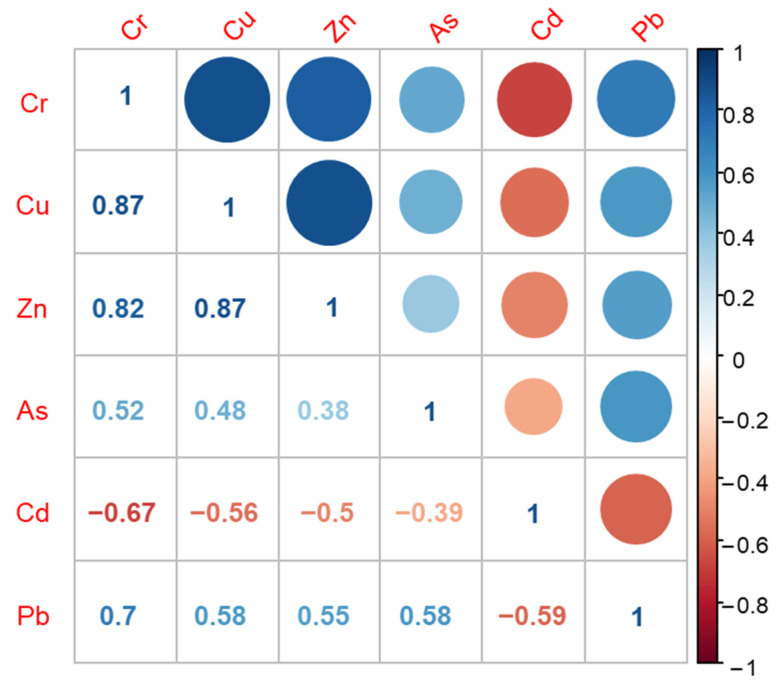
Pearson correlation analysis of heavy metals in the Tianjin coastal area.

As shown in Figure 6, significant correlations were found between each element (including Cr, Cu, Zn, As, Cd, and Pb) (*p* < 0.01). Moreover, Cr showed a significant positive correlation with Cu, Zn, and Pb (*p* < 0.01), and a negative correlation with Cd (*p* < 0.01). Furthermore, a negative correlation was detected between Cd and the other five heavy metals. This could suggest that Cr, Cu, Zn, As, and Pb had similar sources. Furthermore, a significant positive correlation was found between Cd and *EF* and *I_geo_* (*p* < 0.01), indicating that Cd was mainly affected by anthropogenic activities. As shown in Table 5, Cd mainly comes from land-based discharge. Tianjin City has relatively developed industries, such as steel, metal processing, and coastal aquaculture and port transportation [20,69,70].

**Figure 7:**
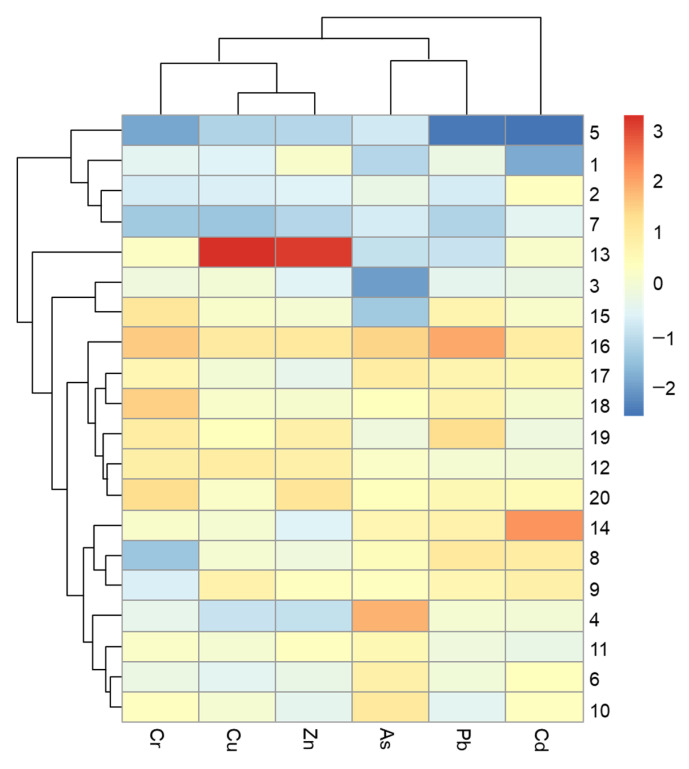
Dual clustering analysis of heavy metals and sampling sites.

Based on the dual clustering analysis results, the horizontal dendrogram figure shows the cluster groups according to heavy metal concentrations in the sediments (Figure 7). All of the variables were segregated into two clusters, with Cluster 1 comprising Cr, Cu, Zn, As, and Pb, and Cluster 2 comprising Cd. The vertical dendrogram was segregated into four clusters. Cluster 1 consisted of four sampling stations (5, 2, 1, and 7), Cluster 2 consisted of sampling site 13, and Cluster 3 contained sampling sites 3 and 15. These sites represented the less polluted region. Cluster 4 contained 13 stations that were located near the river estuary of the Tianjin coastal area, indicating the seriously polluted status in the estuary, influenced by the land-based discharge.

In summary, the Pearson correlation analysis results and the CA analysis results showed a good agreement regarding Cd. In fact, with the amount of emissions caused by big ports, coastal factories, mariculture, and other facilities in the Tianjin harbor increasing, this area has become an important source of heavy metals in Bohai Bay [7,19]. As mentioned above, Cd was mainly derived from anthropogenic activities, including port transportation, mariculture, and metal fabrication [69]. Therefore, the local government could decrease the ecological risk of Cd by reducing the amount of land-based emissions. Furthermore, there were high positive correlation between Cr, Cu, Zn, As, and Pb, implying that they were generated by the same source, and it was verified that these metals also came from the neighboring city [61,71].

## 4. Conclusions

The results of this study showed that the concentrations of dissolved Cu were high in the surface seawater, while the sediments were mainly contaminated by Cd (mean 0.77 mg/kg). A strong pollution status was found for Cd when evaluated using the *EF* and *I_geo_*. The potential ecological risk index (*RI*) implied that the heavy metals in the sediments pose a considerable ecological risk in the study area, and Cd was the major contributor to this potential ecological risk. Pearson correlation and dual cluster analysis suggested that Cd pollution emerges due to anthropogenic activities, including port transportation, mariculture, and metal fabrication along this coastal area. Therefore, it is necessary to control Cd contamination in the future to improve the quality of the marine environment in Bohai Bay. Meanwhile, further studies of the biological toxicity of Cu in seawater should be carried out to find a means to control the biological toxicity of Cu to the living organisms in Bohai Bay.

## Figures and Tables

**Table 1:** Statistical description of the heavy metal concentrations in surface seawater (µg/L).

Sampling Year	Concentration	Cr	Cu	Zn	As	Cd	Pb
May 2011	Range	–	8.20–17.08	ND-69.57	6.24–12.53	0.07–0.18	0.06–0.37
	Average	–	13.30	10.90	7.80	0.11	0.18
	Standard deviation	–	2.70	19.50	1.40	0.03	0.09
September 2011	Range	0.09–0.36	1.25–3.62	15.91–28.90	–	0.19–0.43	0.14–1.69
	Average	0.22	2.53	22.85	–	0.34	1.00
	Standard deviation	0.08	0.60	2.97	–	0.07	0.40
September 2012	Range	0.25–0.67	4.07–7.67	6.15–68.56	0.81–1.90	0.08–0.26	2.65–7.68
	Average	0.44	5.10	40.73	1.39	0.12	4.38
	Standard deviation	0.13	0.87	17.35	0.27	0.05	1.65
Mean		0.33	6.98	24.83	4.60	0.19	1.85
Seawater quality standard (grade I)		≤50	≤5	≤20	≤20	≤1	≤1
Seawater quality standard (grade II)		≤100	≤10	≤50	≤30	≤5	≤5

**Table 2:** Heavy metal concentrations in the surface seawater in some near-shore areas in China (µg/L).

Area	Cr	Cu	Zn	As	Cd	Pb	References
Tianjin Coastal Area	0.32	6.98	24.83	4.60	0.189	1.852	This study
Jinzhou Bay	–	8.31	31.34	–	1.71	4.05	[42]
Yellow River Estuary	–	2.65	37.67	0.92	0.68	0.51	[44]
Dingzi Bay	1.80	3.88	19.73	1.18	1.55	1.11	[45]
Jiangsu Coastal Arae	–	3.12	9.29	1.83	0.12	0.55	[9]
Yangtze River Estuary	0.28	1.47	8.91	1.94	0.04	0.83	[46]
Pearl River Estuary	–	1.64	13.54	2.55	0.12	1.61	[47]

**Table 3:** Statistical descriptions of the heavy metal concentrations in sediments (mg/kg).

Sampling Year	Concentration	Cr	Cu	Zn	As	Cd	Pb
May 2011	Range	49.66–77.12	18.32–75.94	29.19–185.59	11.98–38.23	0.18–0.34	17.40–27.70
	Average	63.96	34.87	77.57	22.42	0.25	23.43
	Standard deviation	10.17	11.95	34.83	7.34	0.05	2.84
September 2011	Range	30.16–45.33	9.54–21.50	18.19–102.64	2.87–53.49	0.16–0.40	16.21–31.81
	Average	40.33	15.81	46.33	19.53	0.29	24.46
	Standard deviation	4.76	3.22	17.64	12.28	0.07	4.09
September 2012	Range	57.08–68.96	24.71–33.93	74.75–133.20	15.41–28.26	0.23–0.77	20.27–26.22
	Average	63.56	29.88	99.57	19.99	0.58	23.32
	Standard deviation	3.27	2.68	16.79	3.24	0.14	1.39
September 2015	Range	4.64–23.65	1.70–13.15	5.93–35.66	0.87–41.62	0.31–2.63	3.29–20.68
	Average	18.48	7.30	20.22	7.01	2.03	14.61
	Standard deviation	5.13	2.63	6.99	8.43	0.61	3.68
Mean		46.58	21.96	60.92	17.24	0.77	21.45
Sediment quality standard (grade I)		≤80	≤35	≤150	≤20	≤0.5	≤60
Sediment quality standard (grade II)		≤150	≤100	≤350	≤65	≤1.5	≤130

**Table 4:** Heavy metal concentrations in sediments in some near-shore areas in China and other worldwide sites (mg/kg).

Area	Cr	Cu	Zn	As	Cd	Pb	References
Tianjin Coastal Area	46.58	21.96	60.92	17.24	0.77	21.45	This study
Bohai Bay, China	48.80	16.10	50.00	28.40	0.10	19.40	[53]
Laizhou Bay, China	32.69	10.99	50.63	7.10	0.19	13.37	[54]
Liaodong Bay, China	–	26.05	71.93	11.03	0.21	24.22	[55]
Jiangsu Coastal Area, China	37.19	23.51	62.16	12.85	0.15	16.87	[9]
Yangtze River Estuary, China	34.40	19.70	71.50	8.80	0.13	25.80	[46]
Daya Bay, China	30.03	10.09	59.34	7.01	0.04	44.18	[56]
Al-Khobar, Arabian Gulf	51.03	182.97	52.68	1.61	0.23	5.36	[57]
Khouran Straits, Persian Gulf	102.40	15.63	40.94	–	0.14	10.30	[58]
Shadegan Wetland, Iran	–	20.42	13.60	3.40	0.27	–	[59]
Red Sea Coast, Egypt	–	9.43	44.15	–	0.53	11.43	[60]

**Table 5:** Land-based discharge of heavy metals in the North China Sea in 2011, 2012, and 2015 (referring to State Oceanic Administration, China, 2011 [63], 2012 [64], and 2015 [65]).

Year	Cu (t/y)	Pb (t/y)	Zn (t/y)	Cd (t/y)	As (t/y)	Total (t/y)
2011	287	131	439	7	96	960
2012	254	286	786	9	89	1424
2015	120	56	522	10	87	795

## Data Availability

Not applicable.

## Supplementary Materials

The following are available online at https://www.mdpi.com/article/10.3390/ijerph182111243/s1, Figure S1: S1 Spatial distributions of the Enrichment factor (EF) and the Geo-accumulation Index (Igeo) of Cd in sediments of the Tianjin coastal area, Table S1: Sampling times and sampling sites of heavy metals from the Tianjin coastal, Table S2: Enrichment factor (EF) of heavy metals in sediments; Table S3 Geo-accumulation index (Igeo), ecological risk coefficient (Ei) and RI of heavy metals in sediments.
