# Supplementary material for: The Pollution Status of Heavy Metals in the Surface Seawater and Sediments of the Tianjin Coastal Area, North China
Source: Int J Environ Res Public Health. 2021 Oct 26;18(21):11243. doi: 10.3390/ijerph182111243 (PMC8582827; doi:10.3390/ijerph182111243)
Supplement: Supplementary file 1 [file ijerph-18-11243-s001.zip › ijerph-1394302-supplementary.pdf]

International Journal of
Environmental Research and Public Health

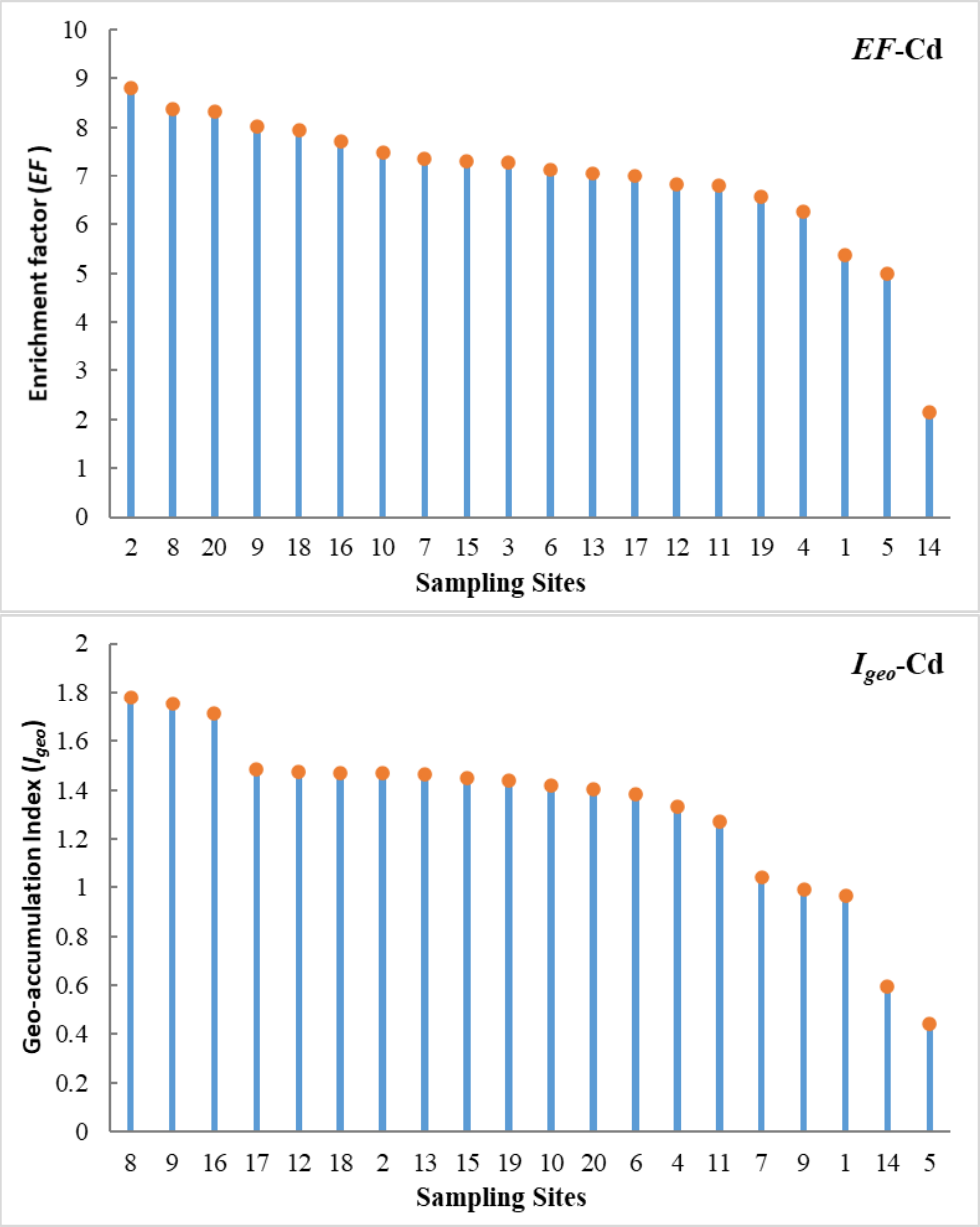

Figure S1. Spatial distributions of the Enrichment factor (*EF*) and the Geo-accumulation Index (I_{geo}) of Cd in sediments of the Tianjin coastal area.

Table S1. Sampling times and sampling sites of heavy metals from the Tianjin coastal area.

Sampling times / Sampling sites	May, 2011		Sep. 2011		Sep. 2012		Sep. 2015	
	Seawater	Sediment	Seawater	Sediment	Seawater	Sediment	Seawater	Sediment
1	√	√	√	√	√	√	×	√
2	√	√	√	√	√	√	×	√
3	√	√	√	√	√	√	×	√
4	√	√	√	√	√	√	×	√
5	√	√	√	√	√	√	×	√
6	√	√	√	√	√	√	×	√
7	√	√	√	√	√	√	×	√
8	√	√	√	√	√	√	×	√
9	√	√	√	√	√	√	×	√
10	√	√	√	√	√	√	×	√
11	√	√	√	√	√	√	×	√
12	√	√	√	√	√	√	×	√
13	√	√	√	√	√	√	×	√
14	√	√	√	√	√	√	×	√
15	√	√	√	√	√	√	×	√
16	√	√	√	√	√	√	×	√
17	√	√	√	√	√	√	×	√
18	√	√	√	√	√	√	×	√
19	√	√	√	√	√	√	×	√
20	√	√	√	√	√	√	×	√

Table S2. Enrichment factor (*EF*) of heavy metals in sediments.

Sampling Year	Stations	*EF* (Enrichment Factor)					
		Cr	Cu	Zn	As	Cd	Pb
May, 2011	1	0.80	0.85	0.80	1.16	1.25	0.94
	2	0.78	0.80	0.43	0.70	1.23	1.02
	3	0.83	1.00	0.57	0.69	1.37	1.11
	4	0.80	0.77	0.56	1.11	1.18	1.06
	5	0.83	0.84	0.50	1.05	1.34	1.05
	6	0.78	0.83	0.72	1.33	1.23	1.14
	7	0.86	0.72	0.40	1.13	1.37	1.04
	8	0.74	1.28	0.92	1.80	2.11	1.35
	9	0.72	1.27	0.89	1.85	2.10	1.38
	10	0.81	1.01	0.60	1.63	1.36	0.95
	11	0.81	0.97	0.90	1.32	1.39	0.95
	12	0.83	1.16	1.03	1.14	1.40	0.97
	13	0.94	2.67	2.28	1.13	2.11	1.08
	14	0.79	0.88	0.67	0.71	1.26	0.94
	15	0.86	0.87	0.60	0.75	1.19	0.93
	16	0.76	0.92	0.65	1.09	1.29	0.85
	17	0.78	0.87	0.74	1.19	1.32	1.03
	18	0.82	0.85	0.58	0.66	1.12	0.89
	19	0.84	0.92	0.89	1.10	1.48	1.10

	20	0.88	0.98	1.11	1.33	1.60	1.08
Sep. 2011	1	1.17	1.06	1.14	0.33	3.60	2.59
	2	1.11	1.31	1.29	2.84	4.70	2.35
	3	1.10	0.88	0.91	0.32	2.95	2.01
	4	1.00	0.90	0.39	5.73	3.60	2.19
	5	1.26	1.22	1.24	2.08	2.86	2.18
	6	1.07	0.96	0.65	2.93	3.43	2.08
	7	1.17	0.96	0.96	1.95	2.79	2.12
	8	0.84	0.63	0.73	2.29	4.58	2.23
	9	1.14	1.18	1.13	2.20	4.66	2.25
	10	1.11	0.79	0.74	2.96	2.96	1.98
	11	1.13	0.81	0.92	3.06	2.64	2.55
	12	1.09	0.97	0.89	1.84	3.31	2.07
	13	1.08	0.91	2.23	0.61	2.98	2.00
	14	1.07	0.98	0.97	0.51	3.03	2.18
	15	1.10	1.02	1.05	0.45	3.82	2.69
	16	1.09	0.99	1.26	3.04	3.97	2.68
	17	1.08	0.96	0.79	2.72	3.25	2.29
	18	1.09	0.89	0.75	3.16	3.39	2.40
	19	1.16	1.13	1.03	1.68	3.42	2.71
	20	1.09	1.01	1.04	2.10	3.05	2.43
Sep. 2015	1	0.39	0.38	0.80	0.33	11.25	1.65
	2	0.35	0.26	0.28	0.26	20.52	0.79
	3	0.37	0.34	0.30	0.22	17.48	0.89
	4	0.35	0.28	0.27	0.26	14.03	0.96
	5	0.36	0.30	0.56	0.27	10.75	0.89
	6	0.35	0.30	0.31	0.51	16.72	0.91
	7	0.36	0.30	0.54	0.45	17.90	1.14
	8	0.37	0.50	0.42	0.49	18.46	1.24
	9	0.39	0.50	0.48	0.30	17.33	0.94
	10	0.37	0.33	0.29	0.56	18.15	0.97
	11	0.36	0.34	0.25	0.41	16.38	1.03
	12	0.35	0.30	0.27	0.45	15.72	0.89
	13	0.34	0.27	0.22	0.26	16.05	0.74
	14	0.34	0.31	0.17	6.00	—	2.12
	15	0.35	0.27	0.25	0.50	16.89	0.81
	16	0.36	0.32	0.24	0.29	17.84	1.09
	17	0.35	0.29	0.26	0.57	16.40	0.77
	18	0.38	0.30	0.30	0.47	19.31	1.03
	19	0.36	0.31	0.27	0.27	14.78	1.01
	20	0.36	0.30	0.26	0.48	20.29	0.82

Table S3. Geo-accumulation index (I_{geo}), ecological risk coefficient (E_i) and RI of heavy metals in sediments.

Sampling Year	Stations	I_{geo} (Geo-Accumulation Index)						E_i (Ecological Risk Coefficient)						*RI*
		Cr	Cu	Zn	As	Cd	Pb	Cr	Cu	Zn	As	Cd	Pb	
May, 2011	1	-0.47	-0.39	-0.47	0.06	0.17	-0.24	2.16	5.74	1.08	15.65	50.74	6.36	81.73
	2	-0.75	-0.72	-1.62	-0.91	-0.10	-0.37	1.78	4.56	0.49	7.98	41.91	5.79	62.52

	3	-0.61	-0.35	-1.15	-0.88	0.11	-0.19	1.96	5.87	0.67	8.13	48.53	6.57	71.73
	4	-0.69	-0.75	-1.20	-0.22	-0.13	-0.29	1.86	4.47	0.65	12.84	41.17	6.13	67.12
	5	-0.76	-0.74	-1.50	-0.42	-0.07	-0.42	1.77	4.48	0.53	11.21	42.97	5.59	66.55
	6	-0.75	-0.66	-0.86	0.01	-0.10	-0.22	1.78	4.74	0.82	15.12	41.91	6.46	70.83
	7	-0.86	-1.10	-1.95	-0.46	-0.18	-0.58	1.66	3.49	0.39	10.91	39.71	5.03	61.17
	8	-0.77	0.02	-0.46	0.51	0.74	0.09	1.75	7.61	1.09	21.34	75.22	8.00	115.02
	9	-0.85	-0.03	-0.55	0.51	0.69	0.09	1.66	7.33	1.03	21.35	72.79	7.97	112.13
	10	-0.24	0.08	-0.69	0.76	0.51	-0.01	2.54	7.93	0.93	25.48	63.97	7.44	108.29
	11	-0.23	0.03	-0.07	0.48	0.56	0.00	2.56	7.67	1.43	20.86	66.18	7.52	106.22
	12	-0.36	0.13	-0.04	0.10	0.40	-0.13	2.33	8.21	1.46	16.08	59.43	6.86	94.37
	13	-0.56	0.95	0.72	-0.30	0.60	-0.36	2.03	14.46	2.47	12.20	68.38	5.83	105.37
	14	-0.44	-0.28	-0.67	-0.60	0.23	-0.18	2.22	6.18	0.94	9.89	52.94	6.61	78.78
	15	-0.23	-0.21	-0.76	-0.42	0.23	-0.12	2.56	6.49	0.89	11.19	52.94	6.89	80.96
	16	-0.26	0.02	-0.47	0.27	0.51	-0.08	2.51	7.59	1.08	18.14	63.97	7.08	100.38
	17	-0.52	-0.36	-0.60	0.08	0.23	-0.12	2.09	5.84	0.99	15.90	52.94	6.89	84.65
	18	-0.22	-0.17	-0.72	-0.53	0.23	-0.11	2.57	6.67	0.91	10.38	52.94	6.97	80.43
	19	-0.36	-0.22	-0.27	0.03	0.46	0.03	2.34	6.44	1.25	15.34	61.76	7.65	94.78
	20	-0.26	-0.09	0.08	0.34	0.60	0.04	2.51	7.03	1.58	18.93	68.38	7.71	106.14
Sep. 2011	1	-1.06	-1.21	-1.10	-2.90	0.56	0.09	1.44	3.25	0.70	2.01	66.24	7.96	81.60
	2	-1.11	-0.87	-0.90	0.24	0.97	-0.03	1.39	4.10	0.80	17.76	87.97	7.32	119.35
	3	-1.19	-1.51	-1.46	-2.97	0.23	-0.32	1.31	2.64	0.54	1.91	52.94	5.99	65.34
	4	-1.27	-1.43	-2.63	1.25	0.58	-0.14	1.24	2.79	0.24	35.66	67.14	6.82	113.89
	5	-1.12	-1.18	-1.15	-0.40	0.06	-0.33	1.38	3.32	0.68	11.37	46.84	5.95	69.53
	6	-1.17	-1.32	-1.88	0.29	0.51	-0.20	1.33	3.00	0.41	18.34	64.29	6.51	93.88
	7	-1.29	-1.58	-1.57	-0.55	-0.04	-0.43	1.22	2.50	0.50	10.22	43.90	5.56	63.91
	8	-1.55	-1.97	-1.76	-0.11	0.89	-0.15	1.03	1.91	0.44	13.90	83.50	6.77	107.55
	9	-1.10	-1.05	-1.11	-0.16	0.93	-0.12	1.40	3.61	0.70	13.47	85.60	6.90	111.68
	10	-1.52	-2.01	-2.09	-0.10	-0.10	-0.68	1.05	1.86	0.35	14.00	41.95	4.68	63.90
	11	-1.58	-2.05	-1.87	-0.14	-0.35	-0.40	1.01	1.82	0.41	13.65	35.36	5.70	57.94
	12	-1.04	-1.21	-1.34	-0.29	0.56	-0.12	1.45	3.24	0.59	12.23	66.14	6.91	90.57
	13	-1.18	-1.42	-0.13	-2.01	0.29	-0.29	1.33	2.80	1.37	3.72	54.93	6.15	70.30
	14	-1.00	-1.13	-1.14	-2.07	0.50	0.03	1.50	3.43	0.68	3.56	63.84	7.65	80.68
	15	-1.10	-1.21	-1.18	-2.39	0.69	0.18	1.40	3.23	0.66	2.87	72.65	8.52	89.34
	16	-1.00	-1.14	-0.80	0.48	0.86	0.29	1.50	3.41	0.86	20.86	81.78	9.19	117.60
	17	-0.99	-1.15	-1.43	0.35	0.61	0.10	1.51	3.37	0.56	19.08	68.45	8.04	101.00
	18	-1.04	-1.33	-1.57	0.50	0.60	0.10	1.46	2.99	0.51	21.16	68.04	8.04	102.20
	19	-1.04	-1.08	-1.21	-0.51	0.51	0.18	1.46	3.54	0.65	10.51	64.23	8.48	88.86
	20	-1.03	-1.15	-1.11	-0.09	0.45	0.12	1.47	3.38	0.70	14.09	61.58	8.17	89.39
Sep. 2012	1	-0.56	-0.52	-0.26	-0.17	1.69	-0.19	2.04	5.23	1.25	13.29	144.90	6.57	173.28
	2	-0.54	-0.56	-0.32	-0.12	1.53	-0.22	2.07	5.10	1.20	13.85	130.15	6.46	158.83
	3	-0.50	-0.25	-0.22	-0.49	0.17	-0.17	2.11	6.31	1.29	10.68	50.74	6.68	77.81
	4	-0.50	-0.45	-0.22	-0.06	1.54	-0.20	2.13	5.50	1.29	14.40	130.45	6.53	160.29
	5	-0.66	-0.49	-0.59	-0.16	1.17	-0.36	1.90	5.33	1.00	13.41	101.47	5.85	128.97
	6	-0.45	-0.33	-0.10	0.00	1.57	-0.18	2.20	5.95	1.40	15.02	133.46	6.62	164.65
	7	-0.58	-0.42	-0.59	-0.28	1.12	-0.24	2.01	5.61	1.00	12.36	98.07	6.37	125.41
	8	-0.63	-0.67	-0.36	-0.55	1.92	-0.21	1.94	4.70	1.17	10.27	170.51	6.48	195.07
	9	-0.60	-0.60	-0.30	-0.47	1.87	-0.19	1.98	4.93	1.22	10.86	164.93	6.60	190.52

	10	-0.54	-0.50	-0.19	-0.34	1.78	-0.15	2.06	5.31	1.31	11.87	154.28	6.75	181.58
	11	-0.49	-0.40	-0.08	-0.15	1.71	-0.13	2.13	5.70	1.41	13.55	147.33	6.86	176.98
	12	-0.45	-0.32	-0.01	0.02	1.68	-0.13	2.20	6.03	1.49	15.26	144.00	6.87	175.85
	13	-0.39	-0.23	-0.01	0.09	1.56	-0.17	2.29	6.40	1.49	16.00	132.35	6.66	165.20
	14	-0.45	-0.26	-0.57	-0.47	1.04	-0.04	2.20	6.27	1.01	10.82	92.65	7.28	120.22
	15	-0.50	-0.36	-0.06	-0.42	1.46	-0.09	2.12	5.84	1.43	11.17	123.53	7.06	151.16
	16	-0.43	-0.22	0.24	0.33	1.86	0.01	2.22	6.46	1.78	18.84	163.24	7.57	200.11
	17	-0.51	-0.42	-0.52	-0.13	1.48	-0.09	2.11	5.61	1.05	13.71	125.74	7.03	155.24
	18	-0.38	-0.31	0.10	-0.11	1.72	-0.05	2.30	6.05	1.60	13.86	147.79	7.23	178.84
	19	-0.46	-0.29	0.14	0.04	1.58	-0.09	2.18	6.14	1.65	15.37	134.43	7.06	166.83
	20	-0.46	-0.50	0.01	-0.34	0.86	-0.28	2.19	5.29	1.51	11.89	81.62	6.17	108.66
Sep. 2015	1	-3.41	-3.45	-2.37	-3.65	1.44	-1.32	0.28	0.69	0.29	1.19	122.43	3.00	127.88
	2	-2.40	-2.83	-2.73	-2.85	3.47	-1.22	0.57	1.05	0.23	2.08	499.41	3.22	506.56
	3	-2.11	-2.22	-2.40	-2.84	3.46	-0.84	0.70	1.61	0.28	2.09	494.42	4.19	503.30
	4	-1.98	-2.31	-2.36	-2.39	3.34	-0.52	0.76	1.51	0.29	2.87	456.02	5.22	466.68
	5	-4.28	-4.54	-3.65	-4.69	0.61	-2.98	0.15	0.32	0.12	0.58	68.48	0.95	70.61
	6	-2.04	-2.25	-2.20	-1.47	3.56	-0.63	0.73	1.57	0.33	5.42	530.15	4.83	543.04
	7	-2.36	-2.61	-1.78	-2.05	3.27	-0.71	0.58	1.23	0.44	3.62	432.98	4.58	443.43
	8	-2.06	-1.64	-1.89	-1.68	3.57	-0.33	0.72	2.40	0.41	4.68	534.31	5.97	548.49
	9	-1.97	-1.58	-1.66	-2.33	3.52	-0.68	0.77	2.50	0.48	2.99	516.06	4.68	527.47
	10	-2.13	-2.31	-2.50	-1.54	3.48	-0.74	0.69	1.52	0.27	5.18	501.96	4.49	514.10
	11	-2.34	-2.42	-2.90	-2.15	3.16	-0.83	0.59	1.40	0.20	3.37	402.78	4.21	412.56
	12	-2.24	-2.45	-2.58	-1.85	3.27	-0.88	0.64	1.37	0.25	4.17	432.64	4.06	443.13
	13	-2.15	-2.49	-2.79	-2.54	3.41	-1.04	0.68	1.33	0.22	2.58	477.94	3.66	486.41
	14	-3.24	-3.38	-4.25	0.89	—	-0.62	0.32	0.72	0.08	27.75	—	4.90	33.76
	15	-2.19	-2.56	-2.68	-1.67	3.41	-0.98	0.66	1.27	0.23	4.71	478.74	3.81	489.42
	16	-2.02	-2.19	-2.58	-2.32	3.62	-0.41	0.74	1.64	0.25	3.01	555.15	5.65	566.44
	17	-1.93	-2.23	-2.35	-1.22	3.62	-0.80	0.79	1.60	0.29	6.43	551.47	4.31	564.90
	18	-2.32	-2.65	-2.67	-2.02	3.34	-0.89	0.60	1.20	0.24	3.70	455.59	4.06	465.39
	19	-2.15	-2.40	-2.56	-2.58	3.20	-0.67	0.68	1.42	0.25	2.51	412.33	4.71	421.90
	20	-2.15	-2.41	-2.59	-1.72	3.69	-0.94	0.68	1.42	0.25	4.55	579.96	3.90	590.76
